# Supplementary figures and images for: Chromosome splitting of Plasmodium berghei using the CRISPR/Cas9 system
Source: PLoS One. 2022 Feb 24;17(2):e0260176. doi: 10.1371/journal.pone.0260176 (PMC8870493; doi:10.1371/journal.pone.0260176)

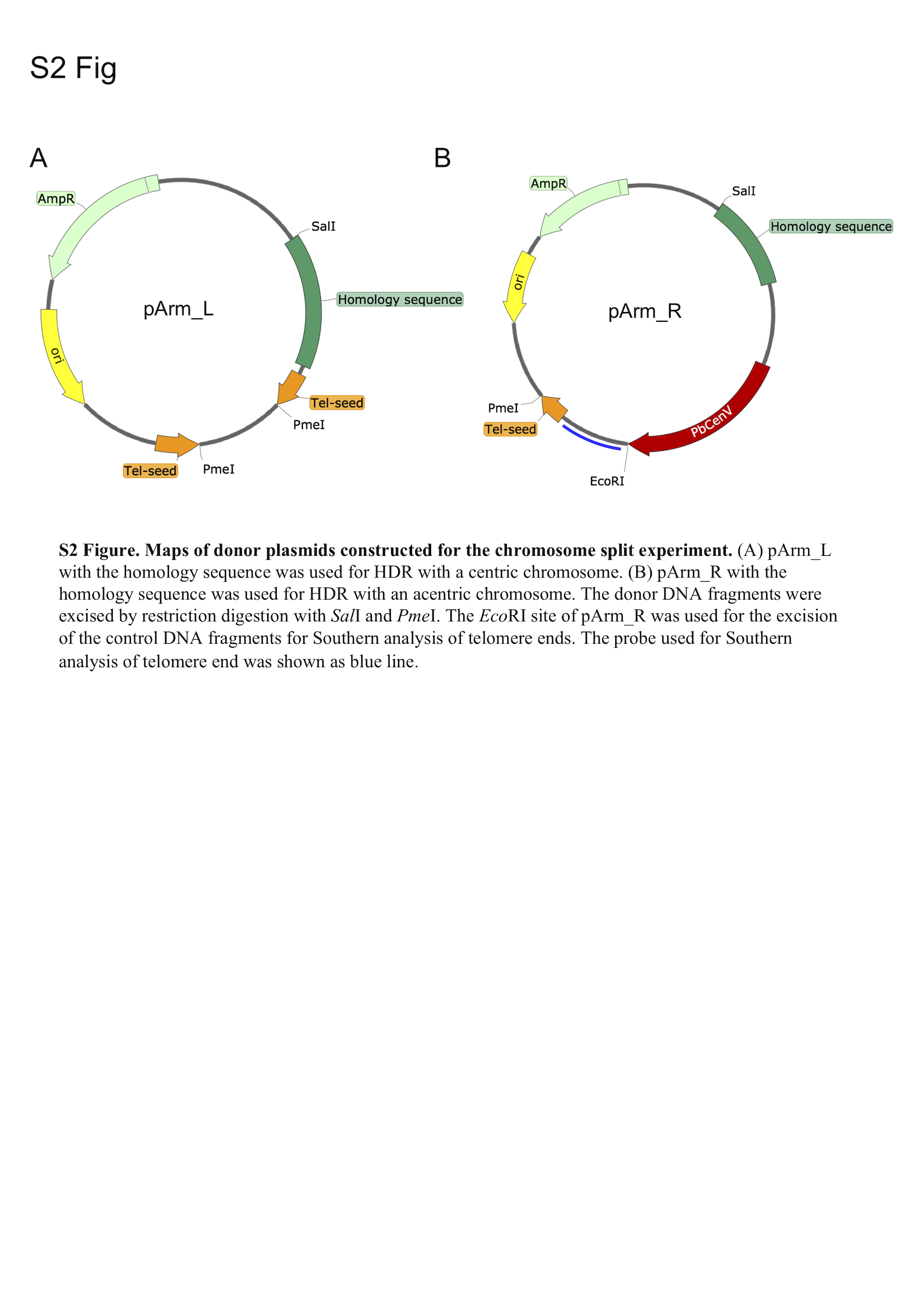

Supplement: S1 Fig — (TIFF) [file pone.0260176.s002.tiff]

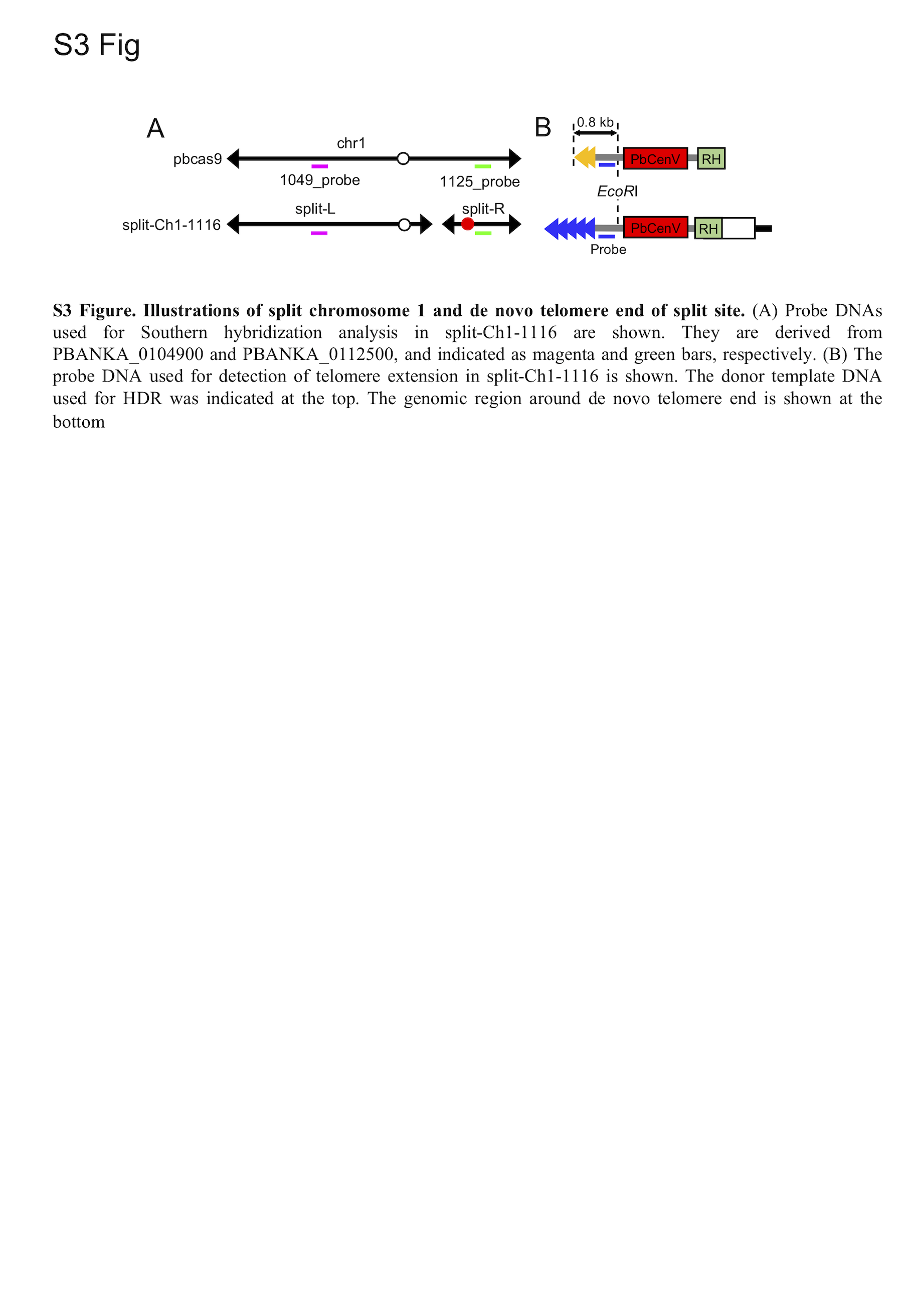

Supplement: S2 Fig — (TIFF) [file pone.0260176.s003.tiff]

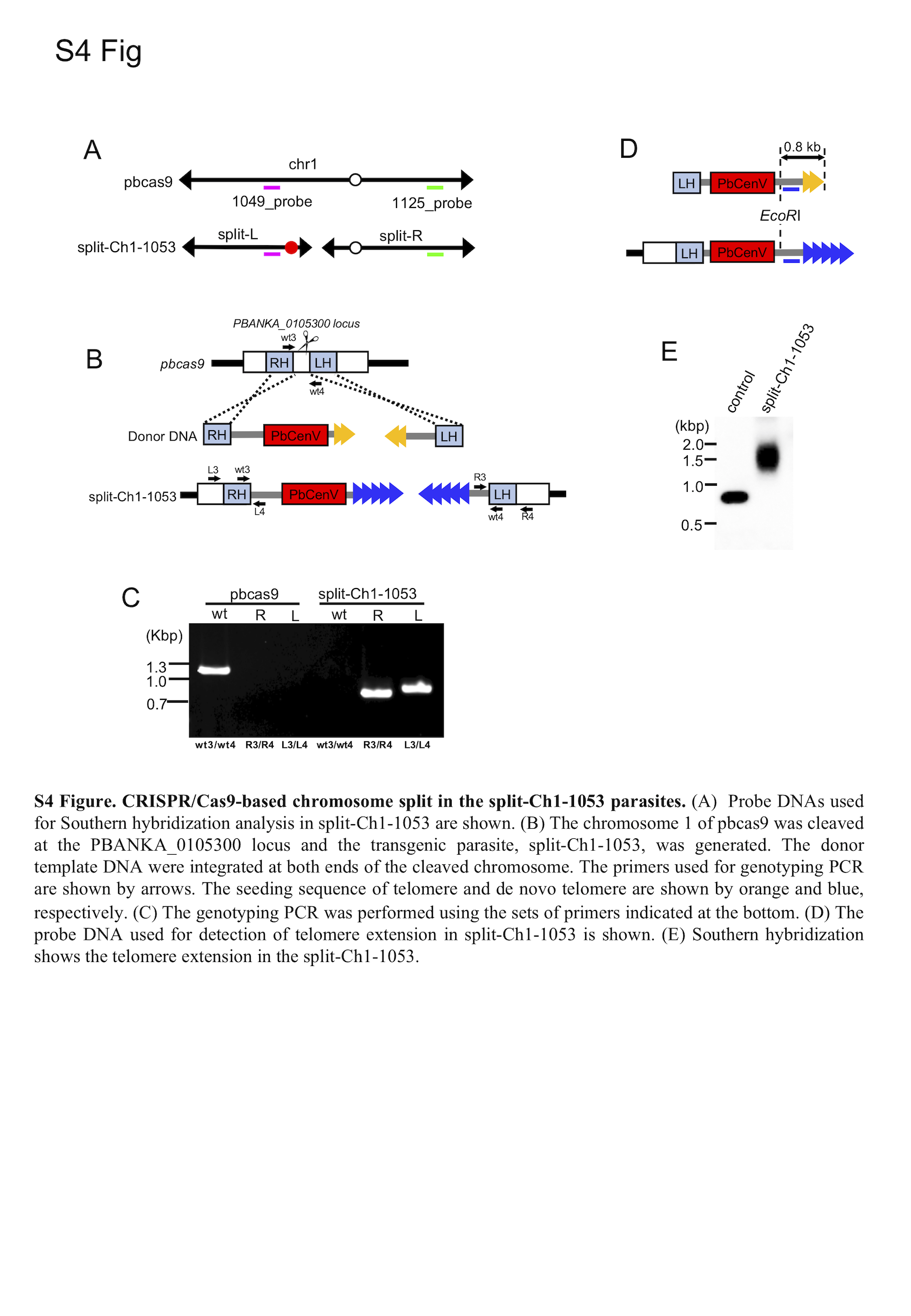

Supplement: S3 Fig — (TIFF) [file pone.0260176.s004.tiff]
