# Supplementary material for: Chromosome splitting of Plasmodium berghei using the CRISPR/Cas9 system
Source: PLoS One. 2022 Feb 24;17(2):e0260176. doi: 10.1371/journal.pone.0260176 (PMC8870493; doi:10.1371/journal.pone.0260176)
Supplement: S1 File — (PDF) [file pone.0260176.s005.pdf]

For Fig. 2B

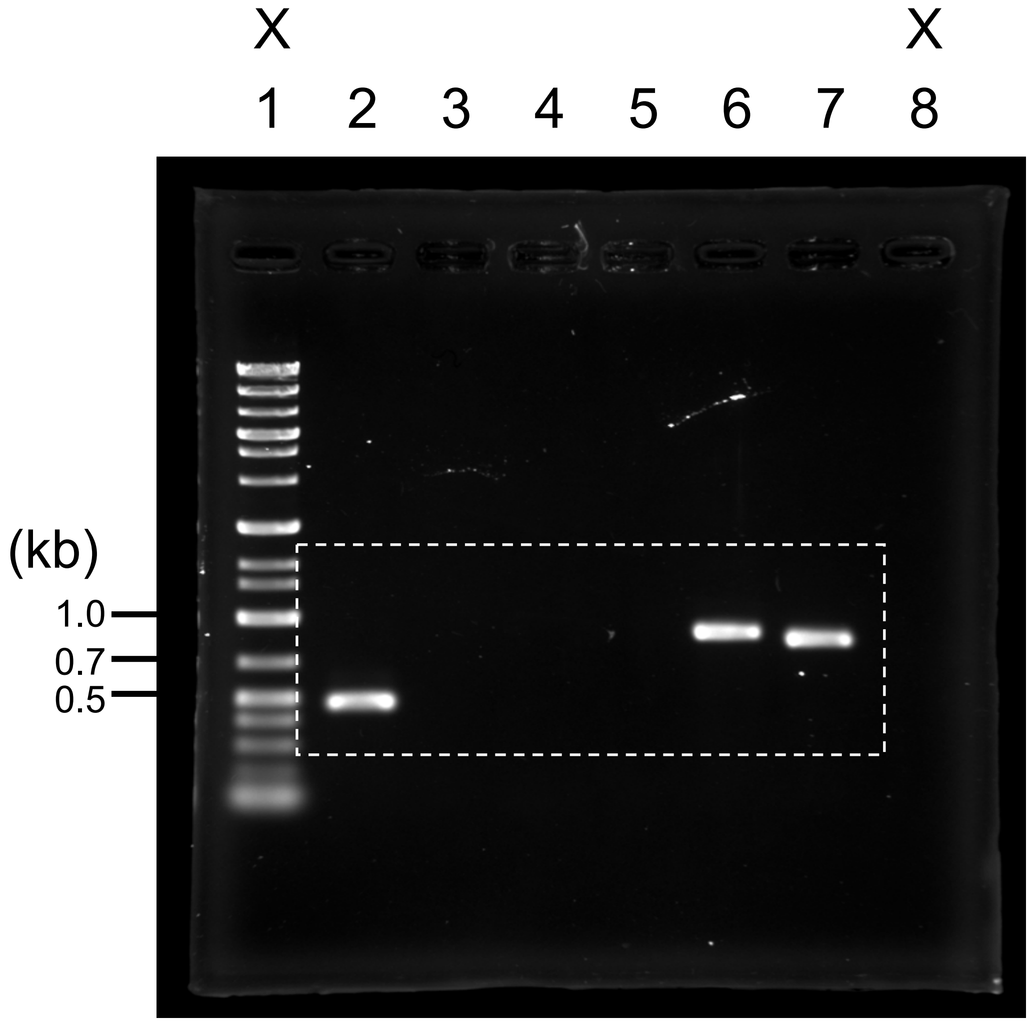

1. Nippongene gene ladder wide 1 (0.1-20 kbp)
2. Template: pbcas9, primers: wt1/wt2
3. Template: pbcas9, primers: R1/R2
4. Template: pbcas9, primers: L1/L2
5. Template: split-Ch1-1116, primers: wt1/wt2
6. Template: split-Ch1-1116, primers: R1/R2
7. Template: split-Ch1-1116, primers: L1/L2
8. Empty well

Photographed with: Chemidoc System (Bio-Rad)

For Fig. 2C-Left

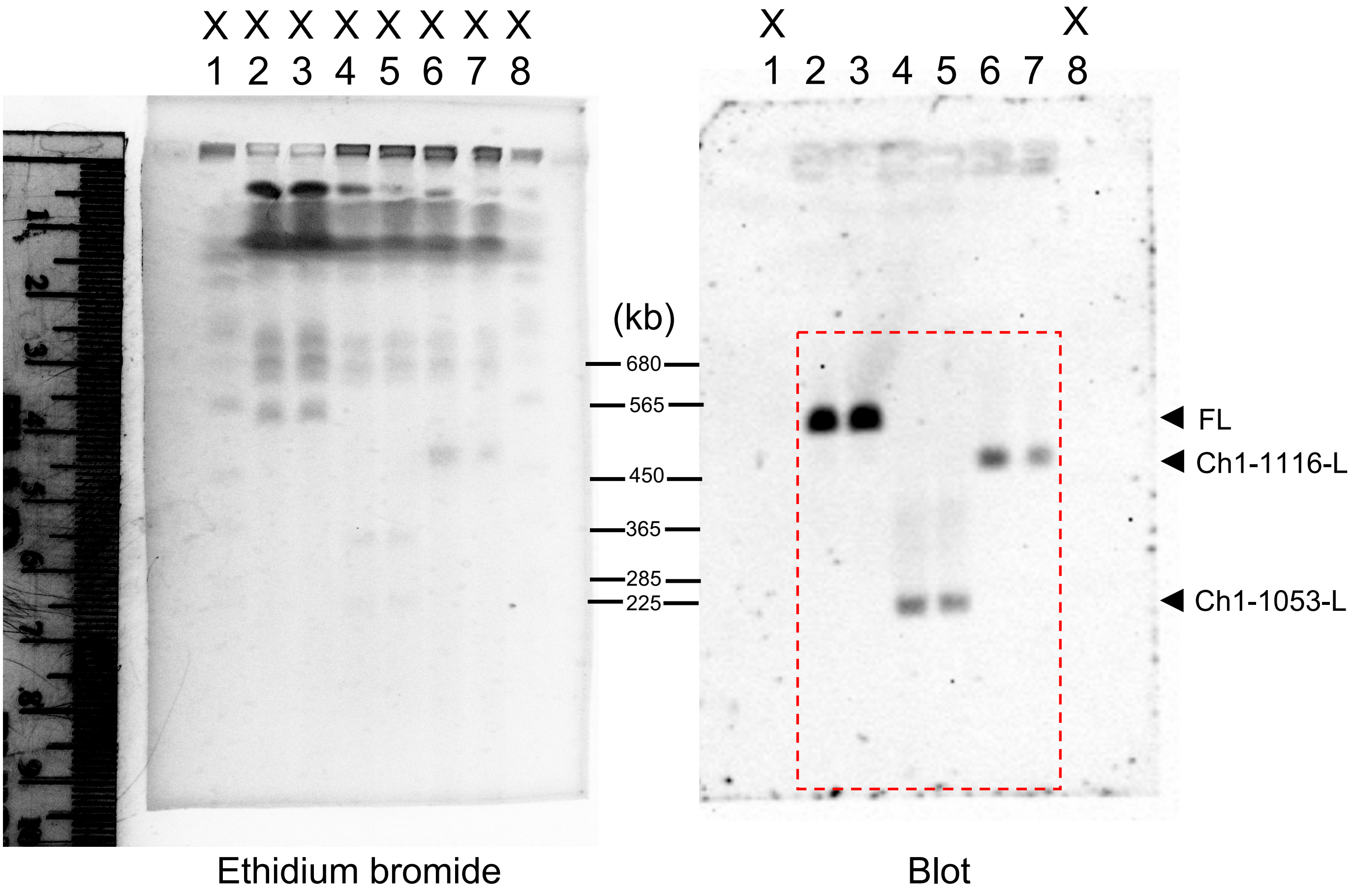

1 and 8: CHEF DNA marker *S. cerevisiae* (BIO-RAD)
2 and 3: pbcas9 genomic DNA
4 and 5: split-Ch1-1053 genomic DNA
6 and 7: split-Ch1-1116 genomic DNA
Probe: 1049_probe
Photographed with: Chemidoc System (Bio-Rad)

For Fig. 2C-Right

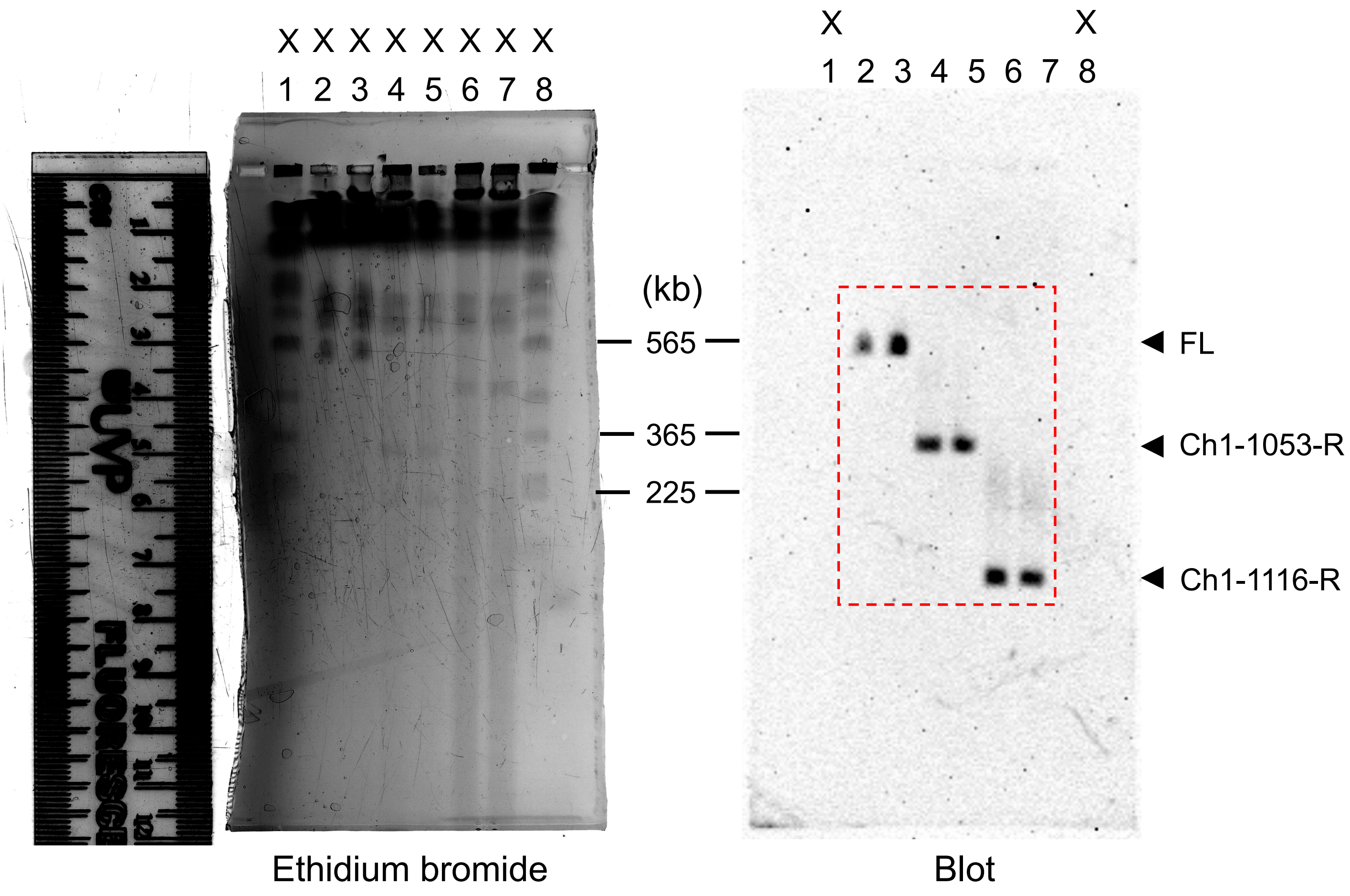

1 and 8: CHEF DNA marker *S. cerevisiae* (BIO-RAD)
2 and 3: pbcas9 genomic DNA
4 and 5: split-Ch1-1053 genomic DNA
6 and 7: split-Ch1-1116 genomic DNA
Probe: 1125_probe
Photographed with: Chemidoc System (Bio-Rad)

For Fig. 2D and Fig. S4E

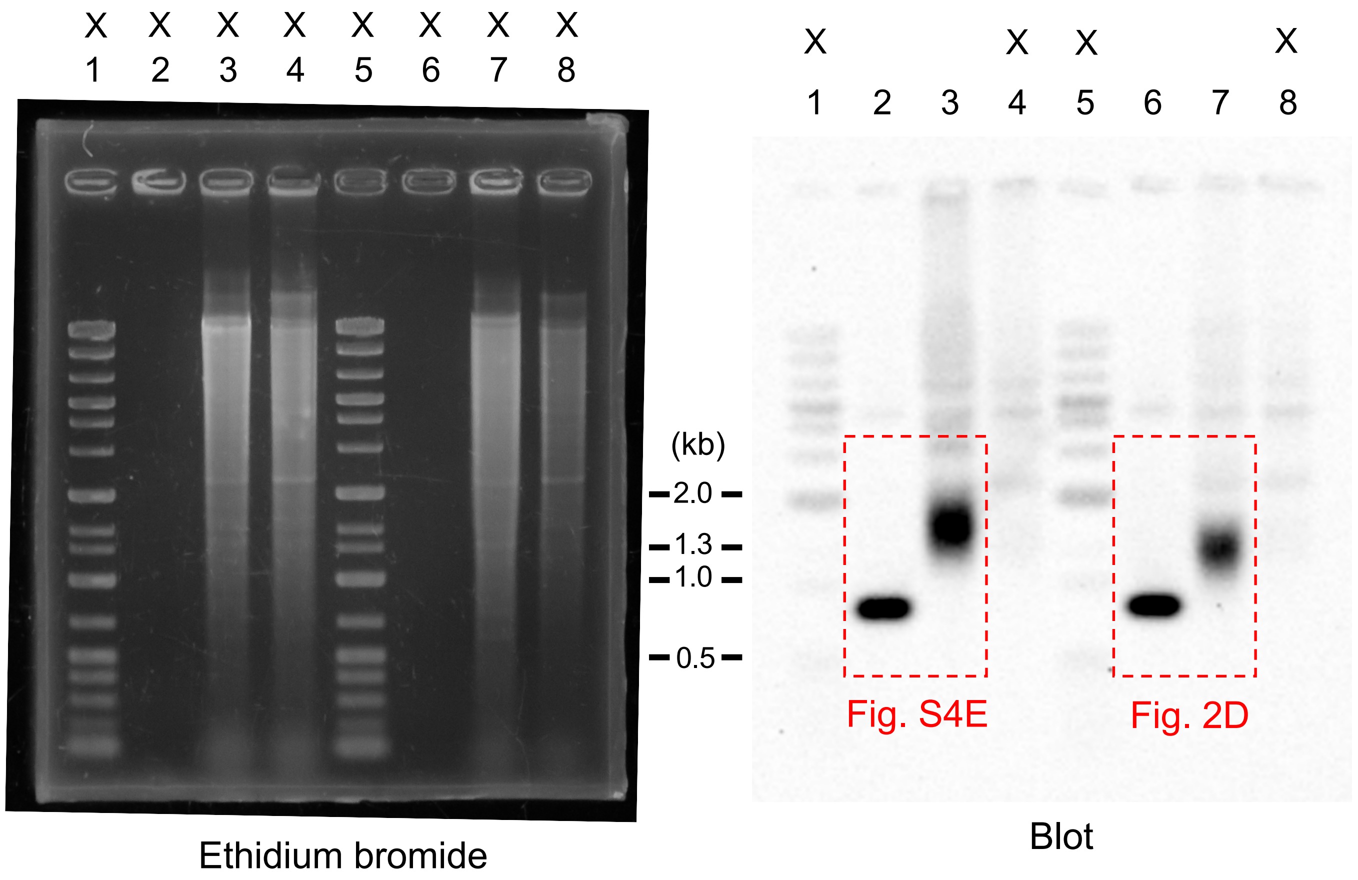

1 and 5: Nippongene gene ladder wide 1 (0.1-20 kbp)
2 and 6: Tel plasmid
3: split-Ch1-1053 genomic DNA
4 and 8: pbcas9 genomic DNA
7: split-Ch1-1116 genomic DNA
Probe: Tel_probe
Photographed with: Chemidoc System (Bio-Rad)

For Fig. S4C

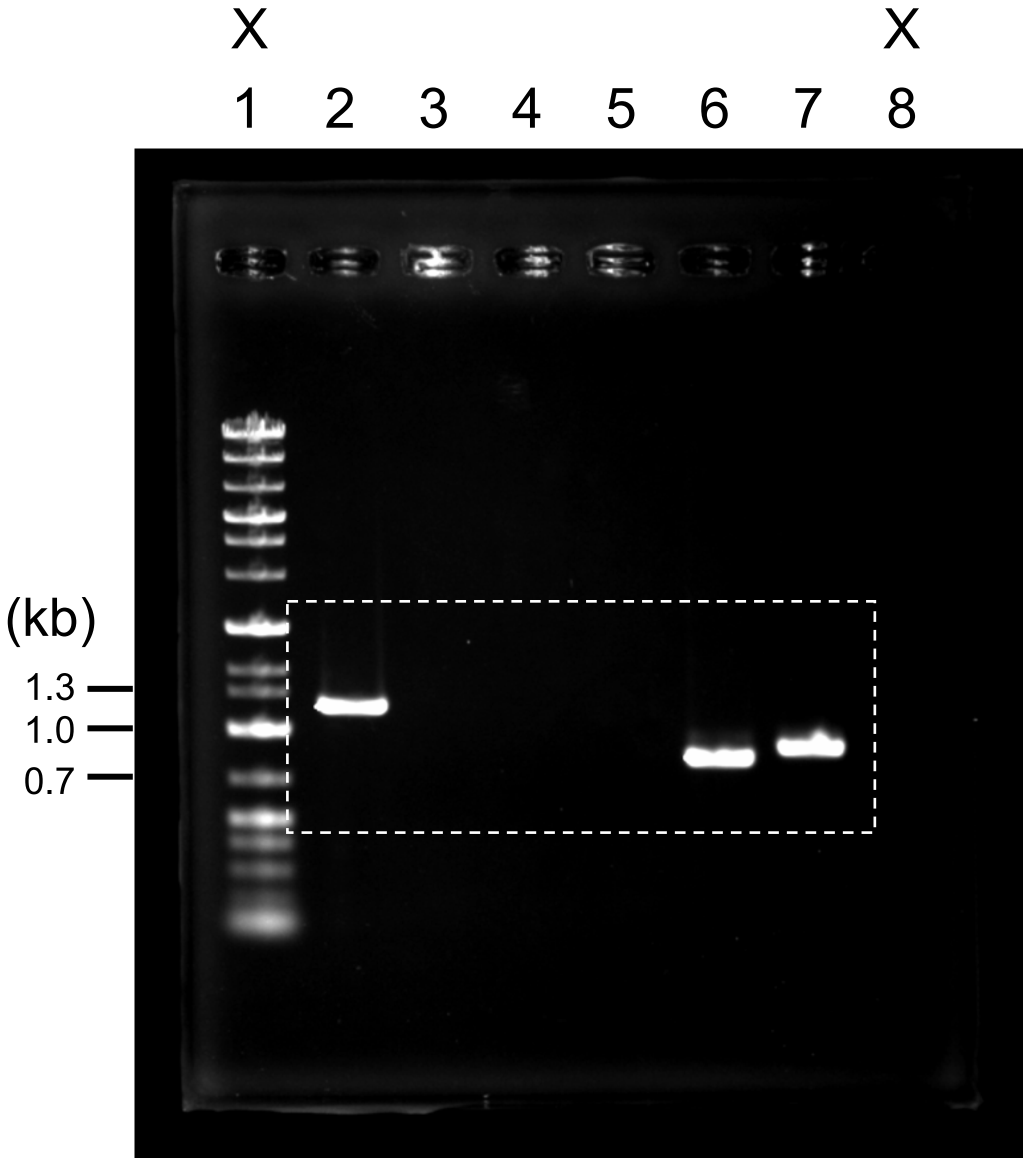

1. Nippongene gene ladder wide 1 (0.1-20 kbp)
2. Template: pbcas9, primers: wt3/wt4
3. Template: pbcas9, primers: R3/R4
4. Template: pbcas9, primers: L3/L4
5. Template: split-Ch1-1053, primers: wt3/wt4
6. Template: split-Ch1-1053, primers: R3/R4
7. Template: split-Ch1-1053, primers: L3/L4
8. Empty Well

Photographed with: Chemidoc System (Bio-Rad)
