# Supplementary material for: Chromosome splitting of Plasmodium berghei using the CRISPR/Cas9 system
Source: PLoS One. 2022 Feb 24;17(2):e0260176. doi: 10.1371/journal.pone.0260176 (PMC8870493; doi:10.1371/journal.pone.0260176)
Supplement: Attachment — Submitted filename: Response to Reviewers_211224__SI.docx [file pone.0260176.s006.docx]

**Journal Requirements**

When submitting your revision, we need you to address these additional requirements.

1. Please ensure that your manuscript meets PLOS ONE's style requirements, including those for file naming.

**Response:**

We have taken a close look at the PLOS ONE style templates and have edited the manuscript to its specifications.

2. To comply with PLOS ONE submissions requirements, in your Methods section, please provide additional information on the animal research and ensure you have included details on (1) methods of sacrifice, (2) methods of anesthesia and/or analgesia, and (3) efforts to alleviate suffering.

**Response**

We have included information on the methods of sacrifice, methods of anesthesia and/or analgesia, and efforts to alleviate suffering as request in the Animal Experiment Section of the materials and methods.

*For all experiments requiring the use of total mouse blood, mice were deeply anesthetized using isoflurane, followed by collection of total blood by cardiac puncture, and euthanized by cervical dislocation immediately after collection. At the end of all mice experiments, euthenization was performed using 70% carbon dioxide and 30% oxygen mixture in a gas chamber, ensuring that mice were completely dead before removal from the chamber.* (P.4,l.82-P.5,l.87)

3. PLOS ONE now requires that authors provide the original uncropped and unadjusted images underlying all blot or gel results reported in a submission’s figures or Supporting Information files.

In your cover letter, please note whether your blot/gel image data are in Supporting Information or posted at a public data repository, provide the repository URL if relevant, and provide specific details as to which raw blot/gel images, if any, are not available.

**Response**

We have attached S5 File including all original PCR and Southern blot pictures and added the description about this supporting file in the cover letter.

4. We note that Figures 3 and 4 in your submission contain copyrighted images. All PLOS content is published under the Creative Commons Attribution License (CC BY 4.0), which means that the manuscript, images, and Supporting Information files will be freely available online, and any third party is permitted to access, download, copy, distribute, and use these materials in any way, even commercially, with proper attribution.

**Response**

All the images contained in the Figures 3 and 4 as well as the other figures were generated from our experiments and hence do not contain any copyrighted material.

5. Thank you for stating the following financial disclosure:  All studies were supported by Grants-in-Aid for Scientific Research (20H03477 and 19K22527 to S.I. 18K07084 and 21K06985 to N.S., and 17H01542 to M.Y.), which were funded by the Japan Society for the Promotion of Science (JSPS: <https://www.jsps.go.jp/>), and also supported by the Japan Agency for Medical Research and Development (AMED: <https://www.amed.go.jp/>) under Grant Number 21jm0210061h0004 (to S.I.), 21wm0325018 (to N.S.) and 21wm0225014 (to N.S.).

Please state what role the funders took in the study.  If the funders had no role, please state: ""The funders had no role in study design, data collection and analysis, decision to publish, or preparation of the manuscript."" 
If this statement is not correct you must amend it as needed.  Please include this amended Role of Funder statement in your cover letter; we will change the online submission form on your behalf.

**Response:**

We described the amended role of funder’s statement in the cover letter.

6. Please review your reference list to ensure that it is complete and correct. If you have cited papers that have been retracted, please include the rationale for doing so in the manuscript text, or remove these references and replace them with relevant current references. Any changes to the reference list should be mentioned in the rebuttal letter that accompanies your revised manuscript. If you need to cite a retracted article, indicate the article’s retracted status in the References list and also include a citation and full reference for the retraction notice.

**Response:**

We carefully checked the reference list. There were no retracted papers

**Reviewer’s Comments**

**Reviewer #1: Summary**

The authors here present a study where they have generated a CRISPR/Cas9-based tool which facilitates the site-specific cleavage of *Plasmoidum berghei* chromosomes. By introduction of additional telomere and centromere elements together with the homology directed repair template, they can thereby generate transgenic parasite lines with split chromosomes that segregate and are faithfully maintained during cell division. The study builds upon improvements to the P. berghei CRISPR/Cas9 system that the some of the authors published earlier. They here report on the on the modifications to the system that enables chromosome splitting, the vector generation and proof of principle experiments where they split P. berghei chromosome 1 in two different (blood-stage) functionally redundant loci. They further report on the normal viability and lack of fitness effect for (one of the?) split chromosome 1 lines.

**Impact and quality** This is a well written manuscript with clearly presented methods and results, and where conclusions are well supported by data. Data is of good quality and is underpinned by appropriate controls. The merit of this study is that it presents a for malaria parasites completely novel system to split chromosomes and generate viable progeny with split chromosomes. This tool in turn, as argued by the authors, makes it possible to study the effect of intra-nuclear spatial location of chromosomes, which the authors argue “may cause a gene expression and epigenetic state change: since telomere ends are invariably anchored to inner nuclear membrane”. They further suggest this will be a particularly useful tool to study the effect of intra-nuclear spatial localization on expression of multigene surface proteins and genes important for sexual development since they localize to the subtelomeric regions of malaria parasite chromosomes.

The impact of the study is limited by that the authors present no evidence for change in intra-nuclear spatial location of split chromosomes (e.g. FISH) or of gene expression (e.g. RNA seq) due to chromosome splitting. Could one not instead argue that no change in gene expression is likely to occur due to chromosome splitting, since no observable change in phenotype is shown for the chromosome 1 split line? The impact of this system on our understanding of malaria parasite biology there for remain hypothetical. This type of data would significantly elevate the impact of the study. Nevertheless, that elevation would likely qualify it for a higher impact journal and I have no reservations in recommending the manuscript for publication as it is, with only minor revisions outlined below.

**Response:**

Thank you for reviewing our manuscript to make it better. The details of the modifications according to your comments are described below.

**Minor revisions**

1. 41 considered recently -> recently considered

**Response**

Thank you for pointing-out this grammatical error. We have modified the phase from “considered recently” to “recently considered” on line 41.

2. 95 this plasmid can be screened -> this plasmid can be selected?

**Response**

We have changed the word “screened” to “selected” on line 98.

3. 103 perhaps will aid reader if you explain centric / acentric chromosome?

**Response**

Thank you for this suggestion. We have included an explanation in parenthesis for both acentric and centric chromosomes. This can be seen from lines 108 to 112 as follows:

This linear DNA fragment was used for HDR with the centric chromosome (Chromosome 1 fragment with the original centromere after cleavage) after cleavage by the Cas9-sgRNA complex. Another DNA fragment, which was used for HDR with the acentric chromosome (Chromosome 1 fragment lacking a centromere after cleavage), was amplified and cloned upstream of both telomere and centromere of pArm_R plasmid (S2 Fig)” (P.5,l.106-P.6,l.110)

4. 277 “… we compared the asexual multiplication of split-Ch1-S parasite to that of pbcas9”. Clarify here if split-Ch1-S is PBANKA_0111600 or PBANKA_0105300 line. Perhaps it is stipulated in methods or elsewhere but would be good to make more obvious. This goes for all figures too, it is not always immediately clear to the reader which split chromosome line you refer to.

**Response**

Thank you for this observation. In order to make it easier to understand which mutant parasite is being referred-to, the labeling for the mutants have been modified. For example, split-Ch1-S has been changed to **split-Ch1-1116**. “1116” indicates that the mutant was produced by splitting the parent parasite at the PBANKA_0111600 locus. Similarly**,** split-Ch1-L has been changed to **split-Ch1-1053.** Also, “1053” indicates that the mutant was produced by splitting the parent parasite at the PBANKA_0105300 locus. This change has been effected throughout the text and figures.

5. Fig S4 Mislabeling, two Panel D instead of E

**Response**

Thank you for pointing-out this mistake. The mislabeled “D” panel has been re-labeled as “E” in the S4 Fig caption.
